# Advances, Challenges, and Applications of Graphene and Carbon Nanotube-Reinforced Engineering Ceramics

**DOI:** 10.3390/nano14231881

**Published:** 2024-11-22

**Authors:** Alaa Almansoori, Katalin Balázsi, Csaba Balázsi

**Affiliations:** 1 Institute for Technical Physics and Materials Science, HUN-REN Centre for Energy Research, Konkoly-Thege Miklós Str. 29-33, 1121 Budapest, Hungary; balazsi.katalin@ek.hun-ren.hu; 2 Technical Institute of Basra, Southern Technical University, AlZubair Str., Basra 42001, Iraq

**Keywords:** ceramics, graphene, carbon nanotubes, functional properties, spark plasma sintering

## Abstract

Engineering ceramics and their composites are widely used owing to their excellent properties, including high wear, corrosion and heat resistance, low friction coefficient, and low thermal conductivity; thus, the current paper presents a comprehensive review of the most common types of engineering ceramics, demonstrating their key properties, advantages, potential applications, and challenges. This paper also provides prevailing methods for tackling the engineering ceramic challenges and maximizing their applicability. This review paper focuses on alumina (Al_2_O_3_), silicon carbide (SiC), zirconia (ZrO_2_), aluminum nitride (AlN), and silicon nitride (Si_3_N_4_), and explores their usability in automotive, aerospace, and tribological applications. Additionally, the incorporation of reinforcing nanomaterials, i.e., graphene and carbon nanotubes or their combination with second-phase reinforcing nanomaterials in these types of ceramics to improve their physico-mechanical properties is also discussed. By strategically adding these reinforcing materials, the brittleness of ceramics can be mitigated, leading to materials that are more suitable for demanding applications in various high-performance industries.

## 1. Introduction

Engineering ceramic materials are well known for their superior properties, such as mechanical properties, including high stiffness, hardness, wear resistance, and thermal properties, for example, their capacity to withstand high temperatures without degrading. Furthermore, engineering ceramics offer preferred properties such as being lightweight and highly resistant to corrosion in harsh environments [1,2]. Thus, ceramics offer a unique combination of diverse properties, making them a suitable class of materials for a broad range of demanding applications. Engineering ceramics, therefore, are extensively used in fields such as manufacturing of batteries [3,4], electronics [5,6,7], and thermal and mechanical parts [8]. Engineering ceramics play a pivotal role in the automotive industry, such as in oxygen sensors, exhaust gas catalysts, and brake systems due to their durability and reliability [9,10]. Moreover, engineering ceramics are capable of withstanding high temperatures and destructive action in an aggressive environment, making them crucial for applications like aerospace engineering, especially in hot structures and thermal protection systems [11,12].

Despite the numerous advantages offered by engineering ceramics, there are still several challenges that need to be addressed, for example, high brittleness, machining difficulties, and costly production [13,14,15]. These drawbacks can limit the range of applications in various industries. However, the use of ceramic composites by incorporating small amounts of nano- or micro-sized materials provides a promising approach to tackling these challenges. Graphene and carbon nanotubes (CNTs) have demonstrated great potential for enhancing fracture and toughness resistance of engineering ceramics as well as reducing their brittleness [16,17,18,19]. Furthermore, the machinability and thermal stability of ceramic composites have been remarkably improved, making them ideal for complex applications [20,21,22,23]. The development of ceramic composites not only eliminates inherent weaknesses but also offers a combination of superior mechanical, thermal, and electrical properties, thereby expanding the potential uses of ceramics in various fields [24,25,26].

Hence, ceramics and their composites have become an outstanding class of materials and are progressively replacing metals and other materials in various fields like grey cast iron in brake systems. This is due to ceramic composites having multifunctional and physiomechanical exceptional properties, for example, high wear resistance and low friction coefficient, which are pivotal for reducing losses in components during rotation and enhancing the durability of components [1,27]. Thus, ceramic composites are continuously used in the production of brake system components like brake discs and pads, which last longer and have less wear compared to other traditional materials [10,28].

Ceramic composites are renowned for their ability to tolerate high wear and corrosion, which highlights their significance in improving the performance and efficiency of mechanical systems in high tribological applications [28,29,30,31,32]. Hence, the use of ceramic composites in these fields helps to reduce energy losses and processes waste and maintenance costs while increasing the overall performance and service life of the equipment. In brief conclusion, the unique and diverse properties of engineering ceramics and their composites make them an ideal choice in modern technologies and industrial applications, enabling innovation and sustainability and improving performance in several sectors [10,15].

The aim of this review paper is to present an overview of recent progress in engineering ceramics and their composites, highlighting their superior mechanical properties, including high wear and corrosion resistance, low friction coefficient, and robustness. In addition, the current paper aims to demonstrate the different kinds of engineering ceramics, especially concentrating on alumina (Al_2_O_3_), silicon carbide (SiC), zirconia (ZrO_2_), aluminum nitride (AlN), and silicon nitride (Si_3_N_4_), and to discuss their advantages and potential applications in fields such as automotive, aerospace, and tribology. In addition, this review undertakes a comprehensive investigation of the role of graphene and carbon nanotubes (CNTs) in ceramic composites.

## 2. Engineering Ceramics

The development of engineering ceramics can be traced back several decades to the need for materials that can withstand high temperatures and harsh environmental conditions. Until the late 20th century, when their potential for engineering purposes was recognized, engineering ceramics were gradually developed, and their applications were limited to traditional applications [33]. Over time, significant progress has been made in the development of advanced technical ceramics, making the promise of structural ceramics much more likely to be fulfilled, except for applications where brittleness and strength are essential. Additionally, technological challenges may exist in the production of objects with complex-shaped and microsized features [34]. However, engineering ceramics are diverse and offer superior properties that make them ideal for a variety of demanding applications. These ceramic materials, such as alumina, silicon carbide, zirconia, and silicon nitride, are known for their exceptional properties, including thermal, mechanical, and chemical properties. For example, engineering ceramics possess high-temperature stability, high wear resistance, lightweight, and ability to withstand harsh environments, making them suitable for a wide array of applications in industries, including aerospace, biomedical, and automotive sectors [14,35,36]. Due to their excellent insulating properties and ability to dissipate energy as heat, as well as their favorable dielectric properties, engineering ceramics are also suited for electronic applications [6,10]. Although ceramics are brittle in nature, and machining and joining them together or to other materials is challenging, advances in processing techniques and a better understanding of their microstructures are driving their expanded uses in various high-performance fields [13,37,38].

### 2.1. Typical Engineering Ceramics and Their Respective Applications

**Figure 1:**
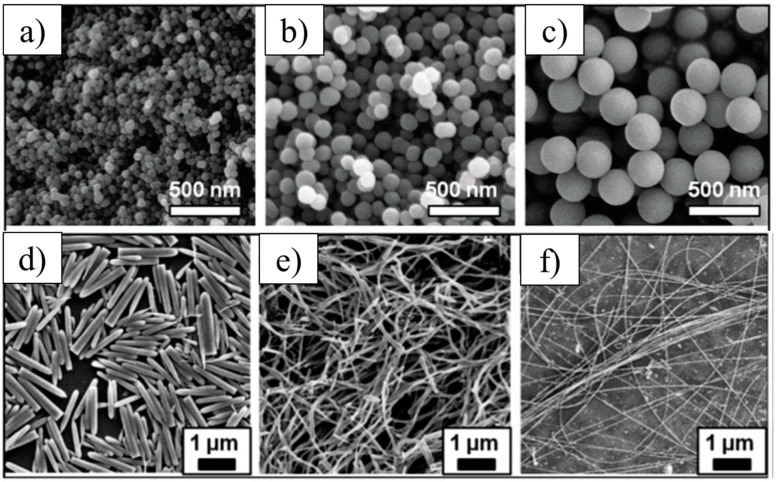
Scanning electron microscopy (SEM) images of SiC nanoparticles with diameters equal to (**a**) 51.3 ± 5.5 nm, (**b**) 92.8 ± 6.6 nm, and (**c**) 278.3 ± 8.2 nm, and rods (**d**) with length of 1.5 ± 0.2 µm, and diameter of 0.20 ± 0.05 µm, (**e**) fibers with length > 10 µm and diameter of 0.18 ± 0.04 µm, and (**f**) fibers with length > 25 µm and diameter = 0.050 ± 0.001 µm [41]. Adapted with permission from [41], published by [RSC Publishing], (2013).

The following are some key types of engineering ceramics:

Silicon Carbide (SiC): Silicon carbide is composed of silicon and carbon atoms joined together by a strong covalent bond. The covalently attached SiC atoms are arranged in a tetrahedral crystal structure to form a very durable ceramic. Although SiC occurs naturally, synthetic SiC can also be obtained when silicon and carbon grains are bonded together using sintering, deposition, reduction, or sol-gel methods [39,40]. Figure 1 displays selected SEM images of synthesized SiC particles in sphere-shaped (a–c) and needle-shaped particles (d–f). The excellent structure of SiC particles and their shapes, shown in Figure 1, play a crucial role in developing nanomaterials used in various engineering fields, making them ideal for high-performance applications.

SiC, therefore, is known for its exceptional hardness and thermal conductivity, making it ideal for high-temperature applications such as components in gas turbines and automotive [39,42,43]. SiC also has excellent chemical resistance and mechanical strength, which are crucial in abrasive environments [9,10].

Alumina (Al_2_O_3_): Aluminum oxide, commonly known as alumina, is a crystalline material produced from aluminum hydroxide, which is extracted from naturally occurring bauxite ores in the Bayer process. Although alumina exists in various crystalline phases, known as transition alumina phases, i.e., γ-, θ-, δ-, η-, and α-alumina, the latter is considered the most thermodynamically stable phase [44]. α-Al_2_O_3_ crystallizes in a corundum structure in which oxygen ions are arranged in an approximate close-packed hexagonal structure surrounding aluminum cations occupying two-thirds of the octahedral sites [45,46]. Thus, α-Al_2_O_3_ has exceptional properties, making it ideal in applications where high-temperature resistance, toughness, and hardness are required, such as cutting tools, grinding media, and high-temperature bearings [47]. Table 1 shows the typical properties of high-density alumina α-Al_2_O_3_.

**Figure 2:**
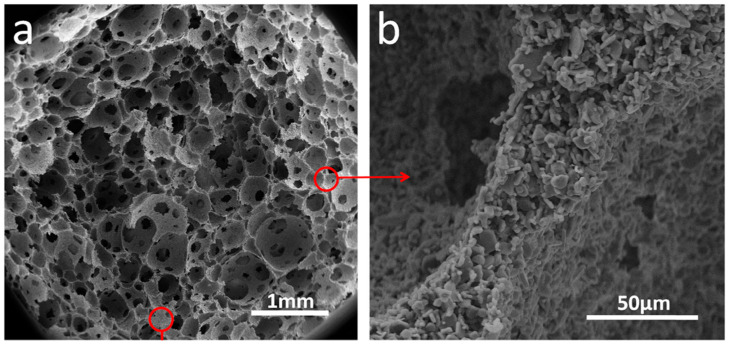
(**a**) SEM images of the pore structure of alumina ceramics, and (**b**) magnified image of pore ridge [50]. Adapted with permission from [50], published by [Elsevier], (2016).

In addition, Alumina is produced from a relatively low-cost process characterized by its excellent thermal stability and resistance to chemical corrosion, making it widely used in components exposed to aggressive chemical environments [47]. Alumina provides a safe option for biomedical implants due to its biocompatibility and high wear resistance. In addition, alumina is a good choice for the production of dielectric substrates [49]. Moreover, Alumina-based ceramics with improved mechanical properties and controllable pore structures have been fabricated using the green biocompatible foaming method [50]. This method is suitable for applications that require highly porous structures such as oil absorption, drug delivery, and tissue engineering. Figure 2 shows an example of porous alumina ceramics with good permeability to water [50].

The ultra-fine α-alumina powder can be synthesized by several methods, including the hydrothermal treatment method, precipitation method, sol-gel method, as well as the Bayer process [51]. In the hydrothermal method, acidic or basic environments can be used to produce alumina ground powder from aluminum salts like aluminum nitrate or aluminum chloride and ammonia solution for the basic route and Tetraethylammonium hydroxide (TEAH) for the acidic route [52], whereas in the precipitation method, the α-alumina powder is synthesized from the aluminum salt (i.e., aluminum nitrate or aluminum chloride) in ammonia solution [51,53]. However, the α-alumina powders synthesized using the hydrothermal approach do not require high temperature or extensive milling [51]. Furthermore, powder made by hydrothermal processes shows less agglomeration, high phase purity, and better chemical purity [54].

**Figure 3:**
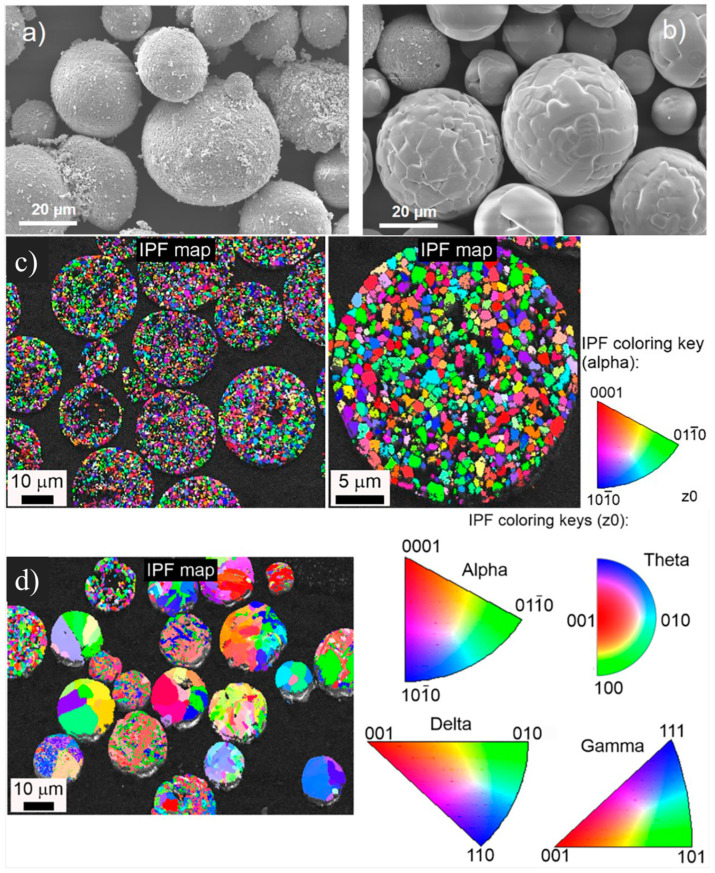
SEM images of typical α-alumina particles before treatment (**a**) and after treatment (**b**). EBSD maps (**c**), inverse pole figure (IPF) map superimposed on the band contrast map of the as-received α-alumina, and (**d**) high-temperature plasma-treated α-alumina [44]. Adapted with copyright from Elsevier under the CC BY.

In addition, the high-temperature plasma treatment followed by the desired cooling rate can optimize the phase structure, resulting in improved end-use properties and expanding the usability of the final products [44]. However, plasma spheroidization can be customized to maintain the most stable α-phase for applications in highly corrosive environments [44]. Figure 3 shows SEM images of untreated and plasma-treated α-alumina (a,b) and EBSD maps of as-received α-alumina (c) and high-temperature plasma-treated α-alumina (d).

**Figure 4:**
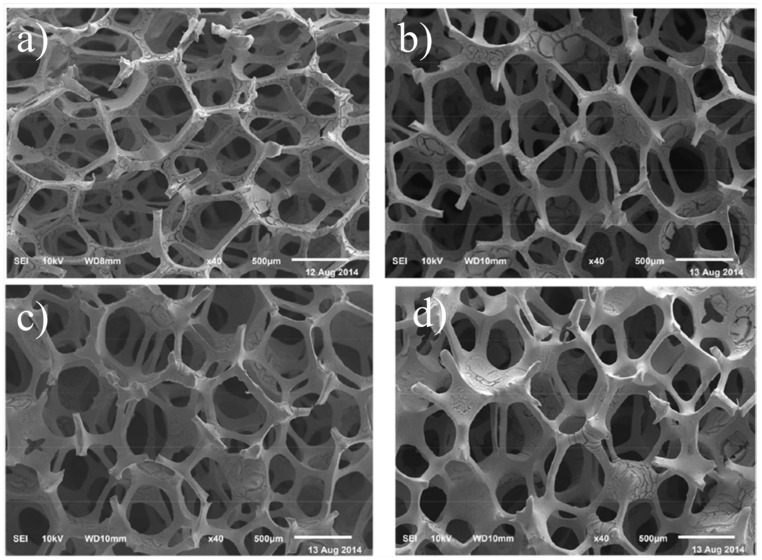
SEM images of porous zirconia scaffold at different sintering cycles (**a**–**d**): one, three, five, and seven sintering cycles, respectively [63]. Reproduced with permission from [63]; published by [IOP Science], (2015).

Zirconia (ZrO_2_), also known as Zirconium dioxide, is a white crystalline ceramic material that occurs naturally as the mineral baddeleyite with a monoclinic crystalline structure. The crystalline content of Zirconia is about 96%–99%, with no glassy phase, exhibiting high fracture toughness and hardness in addition to its superior biocompatibility and aesthetic quality. This makes it suitable for dental implantology and structural applications where durability and precision are required [55,56]. Thus, zirconia is valued in dental applications for the construction of crowns, bridges, and implants, replacing titanium implants [57,58,59]. Moreover, Zirconia nanopowder can be employed as a filling material [60] or nanocoating [61,62] in dentistry and tissue engineering. Owing to zirconia’s excellent strength, hardness, and biocompatibility, it can be employed for the fabrication of porous scaffolds for potential use in bone tissue engineering [63,64,65]. Figure 4a–d show SEM images of fabricated porous scaffolds from zirconia at different sintering cycles: one, three, five, and seven sintering cycles, respectively.

Besides its biocompatibility, Zirconia is renowned for its high strength, hardness, wear resistance, and low coefficient of friction properties, making it an excellent option for applications like cutting tools, refractories, and abrasives [66]. Its excellent resistance to crack propagation further enhances its reliability in mechanical applications [67].

Silicon Nitride (Si_3_N_4_): Silicon nitride is a structural ceramic material crystallized in two major phases, i.e., α- and β-Si_3_N_4_ phases [68,69]. The α-phase and β-phase are the most stable and common phases of Si_3_N_4_ that appear with a trigonal and hexagonal structure, respectively [70]. Moreover, the basic structural units of α- and β-Si_3_N_4_ phases are the same, with tetrahedral bonding (Si atoms) and polyhedral bonding (N atoms) [71]. However, the trigonal α-Si_3_N_4_ may convert to hexagonal β-Si_3_N_4_ at high temperatures of around 1400 °C [70,72]. The third phase, γ-Si_3_N_4_, appears only at high temperatures and pressure [73].

Si_3_N_4_ is one of the most attractive ceramic materials used for applications requiring high temperature and high stress such as gas turbines, diesel engines, automotive, and industrial heat exchangers [74]. Si_3_N_4_ ceramics have a diverse group of superior mechanical properties at high temperatures, including high strength, toughness, and resistance to thermal shock and corrosion at ambient and high temperatures. These properties make it suitable for demanding applications such as engine components, turbochargers, bearings, and cutting tools [75,76]. Si_3_N_4_ also exhibits good wear resistance and low friction, which are advantageous in tribological applications [69,75]. In addition, Si_3_N_4_ offers high biocompatibility, fracture toughness, and hardness properties, making it a good choice for hip and knee joint replacement, dental plants, bone grafts, and scaffolds [70,77]. 

Aluminum Nitride (AlN): Aluminum nitride is one of the ceramic compounds found in the form of nanoparticles with remarkable physiochemical properties, making it the ideal choice in various sectors. It exhibits high thermal conductivity, good thermal stability, and excellent electrical insulation, making it a suitable choice for electronic substrates and heat sinks [78,79]. AlN can also efficiently dissipate heat from electronic equipment, thereby enhancing their performance and reliability [80,81,82].

AlN powder can be synthesized using various methods, such as direct nitridation, carbothermal reduction, and chemical vapor deposition [79,83,84,85]. Direct nitridation is a good choice for synthesizing AlN powder with high purity and better sinterability, while the carbothermal method is recommended as a low-temperature synthesis method [79,84,85].

**Figure 5:**
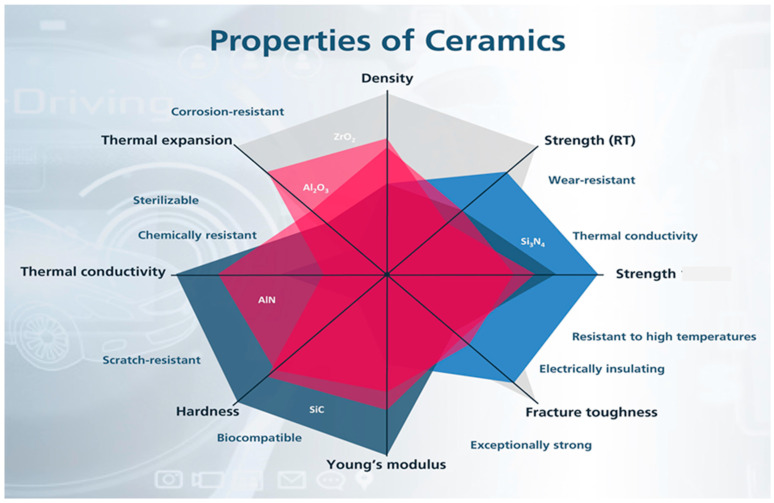
A comparison of the properties of selected ceramics [86].

In summary, each of these selected ceramics has unique thermal, mechanical, and chemical properties that contribute to their widespread use in various high-performance applications. A comparison of the properties of the selected ceramics in this review paper is shown in Figure 5. These examples highlight the significant role of engineering ceramics in advancing technology and improving the performance of various industrial applications. Table 2 briefly demonstrates that engineering ceramics have a wide range of important applications owing to their unique properties. Although engineering ceramics, as discussed earlier in this paper, have a variety of applications, the challenges and limitations associated with their use in engineering applications need to be addressed. For example, their brittleness, high cost, and difficulty of processing make ceramics difficult to use in structural applications, which limits their widespread use; therefore, investigation and testing, including wear and hardness, are essential in determining the properties of ceramics. Additionally, processing methods, such as ball milling and spark plasma sintering, can be analyzed to offer insights into the optimization of ceramic properties for specific applications.

**Table 2:** Summary of selected ceramics and their properties and applications in industry.

Ceramics	Key Properties	Applications
Silicon Carbide (SiC)	Exceptional hardness, high thermal conductivity, chemical resistance, mechanical strength	Gas turbines and automotive [42,43], abrasive materials [9,10], biomedical devices [87].
Alumina (Al_2_O_3_)	High wear resistance, temperature resistance, toughness, hardness, thermal stability, chemical corrosion resistance, biocompatibility.	Cutting tools, grinding media, high-temperature bearings [47], aggressive chemical environment [47], biomedical implants [88], dielectric substrates [49].
Zirconia (ZrO_2_)	High fracture toughness, hardness, thermal expansion, resistance to crack propagation, biocompatibility.	Dental implants, structural applications, bone tissue engineering [57,58,63], nanocoating [61,62].
Silicon Nitride (Si_3_N_4_)	Superior mechanical properties at high temperatures, high strength, toughness, resistance to thermal shock, wear resistance, low friction	Engine components, turbochargers, bearings, and cutting tools [75,76], tribological applications [69,75], joint replacement [70,77].
Aluminum Nitride (AlN)	High thermal conductivity, thermal stability, electrical insulation, heat dissipation performance	Electronic substrates and heat sinks [78,79]

### 2.2. Investigation and Testing of Engineering Ceramics

Hardness Testing: Hardness testing is essential for determining the wear resistance and durability of ceramic materials and their composites [89]. Hardness is essential for applications where ceramic materials are subjected to mechanical stress and surface interactions, such as wear-resistant coatings [90], linings, and components; thus, hardness behavior is crucial for optimizing material performance in environments demanding hard materials such as aerospace, automotive, and biomedical applications [91]. Graphene has shown significant potential for improving the hardness of ceramic materials, such as SiC, for coating purposes by enabling epitaxial growth of graphene on SiC [42]. However, it should be noted that the hardness of the ceramic material itself without a protective coating does not improve with the addition of graphene, as shown in Table 3 and Table 4. Similarly, the addition of CNT could reduce the hardness of ceramic materials such as alumina, attributed to the reduction in density and increase in porosity [92].

### 2.3. Wear Test

With the development of ceramic and composite materials, it is crucial to evaluate their surface properties and substrate-coating combinations, particularly for applications demanding high surface performance. Wear is a major concern in the tribological use of even the hardest materials, including ceramics, during contact with rotating parts or abrasive media, which involves removing material and occurs mostly in the outer surfaces. Therefore, researchers are particularly interested in wear resistance tests to investigate the effects of contact abrasion or erosion on coated and uncoated ceramic components. Owing to their good wear properties, ceramics such as silicon nitride, alumina, and zirconia have been proposed for applications where wear performance is critical; thus, they are preferred for use in industrial or biomedical applications such as cutting tools, turbine blades, and bearings, as well as in hip and knee replacements [47,63,75,93,94]. However, the wear behavior of the main ceramic bulk material can be further improved to increase their application by combining them with secondary-phase nanomaterials to create hybrid materials with improved performance [95]. For example, Yttria-stabilized zirconia (YSZ), zirconia-toughened alumina (ZTA), alumina-toughened zirconia (ATZ), and others are ceramic–ceramic systems that exhibit improved wear performance [95]. Silicon carbide (SiC)/graphene ceramic composites have also been investigated for their potential use in face seals. These composites containing thermally reduced graphene oxide (TRGO), graphene-enhanced silicon carbide (GSiC), and benchmark materials with carbon-reinforcing materials were produced and tested tribologically under water-lubricated conditions. Notably, the results showed that all the produced composites, except graphite, exhibited improved friction and wear performance in an aqueous environment compared to the material without graphene [30]. Thus, it can be concluded that ceramics and ceramic composites show promise in terms of wear performance, making them useful in a wide range of applications that require high wear resistance and biocompatibility for specific uses.

Graphene and CNT integration into ceramic matrices is accomplished using advanced processing techniques. Hot pressing applies heat and pressure at the same time, resulting in densification and reduced chances of agglomeration. Nanomaterials are uniformly distributed in the ceramic matrix through this method, therefore improving the composites’ mechanical properties. For example, a study conducted by Yazdani et al. demonstrated that the tribological performance of graphene/CNT-reinforced Al_2_O_3_ composites produced by hot pressing showed remarkable improvements in friction coefficients along with a promising wear rate reduction [96]. However, hot-pressed graphene/Si_3_N_4_ composites did not improve or improved wear rates only slightly, while the coefficient of friction results showed improvement, especially when 3% graphene was added, as shown in a study by Maria et al. [97].

To produce ceramic nanocomposites, spark plasma sintering (SPS) is widely used. SPS has become a key technology for producing a new generation of advanced materials. Utilizing a pulsed electric current, SPS heats up the material, making it densify quickly at low temperatures compared to other sintering methods. This technique helps to maintain the nano-structure of GNPs and CNTs, which is necessary for maintaining their reinforcement effects [98]. In fact, SPS-processed ceramics possess higher hardness, toughness, and improved electrical conductivity, plus thermal conductivities [99,100]. SPS is particularly effective in enhancing the properties of zirconia (ZrO_2_) and alumina (Al_2_O_3_) composites. Liu et al. (2012) demonstrated that adding graphene platelets to these composites significantly improves their toughness and strength. The rapid sintering process of SPS helps with preserving the integrity of the graphene platelets, thereby enhancing the overall composite performance [101]. Anselmi-Tamburini et al. (2004) conducted a study on the densification of fully stabilized zirconia using SPS. Based on their results, densification is highly influenced by temperature and pressure, but not heating rates and holding time. Thus, they found that SPS could achieve near-theoretical density at a temperature of about 1200 °C. This is attributed to the simultaneous application of pressure and electric current, which enhances the diffusion processes and promotes particle bonding [102]. Table 3 compares the results of the density and hardness of Si_3_N_4_ composites fabricated by hot press and SPS techniques performed by Maria et al. [97].

**Table 3:** Properties of Si_3_N_4_ and graphene/Si_3_N_4_ composite produced by HIP and SPS: apparent density, relative density, Vickers hardness, and VIF toughness [97]. Reproduced with permission from [97]; published by [Elsevier], (2016).

Sintering Method	MLG (wt%)	Apparent Density (g/cm^3^)	Relative Density (%)	HVM0.5 (GPa)	VIF Toughness (MPa·m^1/2^)
HIP	0	3.23	96.52	13.3 ± 0.48	4.7 ± 0.38
	1	3.27	97.94	11.8 ± 0.68	4.2 ± 0.14
	3	2.80	84.66	5.8 ± 0.65	5.7 ± 0.28
SPS	0	3.23	96.54	15.8 ± 0.84	5.1 ± 0.47
	1	3.29	98.52	15.4 ± 0.70	4.9 ± 0.72
	3	3.11	93.84	13.7 ± 0.82	2.7 ± 0.37

## 3. Engineering Ceramics Challenges

Despite the numerous advantages of engineering ceramics, they have significant drawbacks that can limit their applications. One of the primary disadvantages is their intrinsic brittleness, which leads to weak mechanical properties, particularly in terms of toughness. Ceramics are generally hard and resistant to wear but tend to fracture easily under tensile stress or impact, which can compromise their reliability in applications requiring high mechanical strength and toughness [103]. The ceramics’ brittleness originates from their atomic structures, which lack the ability to deform plastically. This means that, unlike metals, ceramics cannot absorb and dissipate energy through plastic deformation, making them prone to catastrophic failure when subjected to stress [38]. This property is particularly detrimental in structural applications where mechanical reliability is critical. In medical applications, brittle failure of ceramic materials can lead to severe clinical complications associated with ceramic crowns and hip replacements, which highlights the importance of fracture toughness of ceramics [14,104].

To overcome this limitation, various strategies have been developed to enhance ceramic toughness and increase their wearability. These strategies involve the addition of a second-phase ceramic material or reinforced materials, such as carbon nanotubes and graphene, to create ceramic composites. These composites can significantly improve fracture toughness by mechanisms such as crack deflection, bridging, and pull-out [14]. In addition, advanced processing techniques, most importantly hot pressing and spark plasma sintering, can improve the microstructural properties of ceramics, resulting in improved mechanical performance [7,26,39]. However, other physio-chemical properties can also be improved to expand applications in various sectors. Hence, improving the toughness, strength, thermal, wear, and corrosion properties of ceramics is pivotal for expanding their uses in various sectors where mechanical reliability is required, like in the aerospace, automotive, and biomedical fields [9,31]. By addressing these challenges, the full potential of engineering ceramics can be realized, thus enabling their applications in more demanding environments, including automotive, aerospace, and medical industries. It could also reduce energy consumption while protecting the environment.

## 4. Improving the Properties of Engineering Ceramics

Improving the properties of engineering ceramics is commonly achieved by developing ceramic composites. These composites improve the mechanical behavior of traditional polycrystalline ceramics, which are typically characterized by high brittleness and low fracture toughness [105,106]. By incorporating small quantities of materials, such as reinforcing phases, the overall properties of ceramics can be significantly enhanced. Ceramic composites are generally created by embedding at least one micro- or nano-sized material and/or reinforcing secondary phase into a ceramic matrix:

(1) The secondary phase can include ceramic micro- or nanoparticles, such as silicon carbide (SiC) and silicon nitride (Si_3_N_4_), which enhance toughness and wear resistance.
(2) The inclusion of carbon nanotubes (CNTs) as a reinforcing material for enhancing toughness and functional properties, such as electrical and thermal conductivity, of ceramic matrices.
(3) Graphene nanoplatelets are another effective reinforcing material that can be used to improve the fracture toughness of ceramics through mechanisms like crack deflection, bridging, and pull-out, leading to increased mechanical performance.
(4) Hybrid composites can be obtained by combining two or more reinforcing materials. For example, CNTs and graphene, or the combination of second-phase nano/micro materials with graphene and CNTs can further enhance the properties of ceramics, resulting in a balance of strength, toughness, and other functional properties.

The mechanical properties of ceramic composites are essentially influenced by the interphase between the ceramic matrix and the reinforcing material. In the case of weak interphase, initiated cracks are more likely to deflect along the interface between the ceramic matrix and the reinforcing phase. This deflection, however, prevents the crack from propagating through the reinforcing phase, maintaining the integrity of the reinforcement and improving the toughness of the composite. Hence, these composites can effectively resist fracture and perform well under mechanical loads [100,107].

In contrast, if the interphase and associated interfaces between the two material phases are too strong, initiated cracks penetrate the reinforcing phase, leading to transgranular fracture. This penetration, as a result, makes the composite brittle, similar to the behavior of pure ceramics, which lack the ability to absorb and dissipate energy during deformation [1]. Therefore, achieving the optimal balance in the matrix–reinforcement interaction plays a key role in enhancing the mechanical performance of ceramic composites [24].

By selecting the right combination of materials (matrices and reinforcements) and optimizing the interactions between them, ceramic composites can achieve excellent mechanical behavior, making them an ideal choice for a wide range of high-performance applications.

### 4.1. Using Nanosized Ceramic Particles as Secondary Phases

The incorporation of second-phase ceramic nanoparticles has remarkably improved the mechanical and functional properties of ceramics. Various nanoparticles, including silicon carbide (SiC), silicon nitride (Si_3_N_4_), titanium carbide (TiC), titanium dioxide (TiO_2_), and zirconium dioxide (ZrO_2_), have been successfully embedded in ceramic matrices such as alumina (Al_2_O_3_), silicon nitride (Si_3_N_4_), and magnesium oxide (MgO) to produce an important class of ceramic nanocomposites [7]. Some of these composite materials include Si_3_N_4_–SiC, Al_2_O_3_–SiC, Al_2_O_3_–Si_3_N_4_, Al_2_O_3_–TiC, Al_2_O_3_–ZrO_2_, and MgO–SiC, which have been extensively studied [108].

Zirconia (ZrO_2_), for example, has been widely investigated as a ceramic composite material, either as a secondary phase or matrix, owing to its exceptional properties. ZrO_2_ exhibits excellent ionic conductivity and superior mechanical properties such as excellent wear resistance, high mechanical strength, and stability at high temperatures [7]. This makes these materials ideal for structural, tribological, and multifunctional applications. For instance, the inclusion of ZrO_2_ particles in Al_2_O_3_ as a second phase, the so-called zirconia-toughened alumina (ZTA), enables ZTA to exhibit higher fracture toughness than an alumina ceramic matrix, but with moderate Vickers hardness [67,109]. Additionally, alumina-toughened zirconia (ATZ) exhibits enhanced fracture toughness when Al_2_O_3_ acts as a secondary phase [14].

Silicon carbide (SiC) nanoparticles have been incorporated as a semiconductive second-phase material in alumina matrices to enhance thermal and electrical conductivities. This combination is beneficial for applications requiring high thermal conductivity as well as durability and resistance to abrasion, such as cutting tools, wear-resistant tools, and gas radiant burners. Considering electrical conductivity, an Al_2_O_3_-SiC composite with moderate electrical conductivity is suited to DC electronic applications [110]. Similarly, silicon nitride (Si_3_N_4_) reinforced with titanium nitride (TiN) nanoparticles improves toughness and thermal shock resistance. This enhancement is critical for applications in high-temperature environments, such as engine components and turbine impellers [111]. Titanium dioxide (TiO_2_) nanoparticles embedded in magnesium oxide (MgO) matrices improve the optical and photocatalytic properties of ceramics, making them suitable for photocatalysis (such as reduction of CO_2_, degradation of CH_3_CHO, and dye degradation) or other applications like dye-sensitized solar cells or nanofluids with high thermal conductivity [112].

These examples demonstrate how incorporating different nanoparticles into ceramic matrices can tailor the properties of the resulting nanocomposites for specific applications. The enhanced properties, including mechanical strength, thermal stability, and functional capabilities, have expanded the utility of ceramics in various high-performance fields.

### 4.2. Using Graphene Nanomaterials to Increase Mechanical and Functional Properties of Ceramics

Graphene is increasingly being used to improve the mechanical properties of ceramic composites due to its exceptional strength and flexibility. In particular, adding small amounts of graphene to ceramics such as silicon nitride (Si_3_N_4_) can significantly enhance fracture resistance [22] or improve tribological behavior [31]. Typically, a graphene content of 2 wt% improves the fracture resistance of Si_3_N_4_ ceramic composites through three primary toughening mechanisms: crack bridging, crack deflection, and pull-out [107,113]. These mechanisms help to prevent crack propagation and enhance the material’s toughness [113].

However, increasing the graphene content beyond this range can sometimes lead to a decrease in mechanical properties. This decline is often attributed to the inhomogeneous distribution of graphene within the matrix or reduced surface interaction between graphene and the ceramic matrix. Ensuring uniform dispersion and optimal interaction is crucial for maximizing the benefits of graphene reinforcement [107,113].

**Figure 6:**
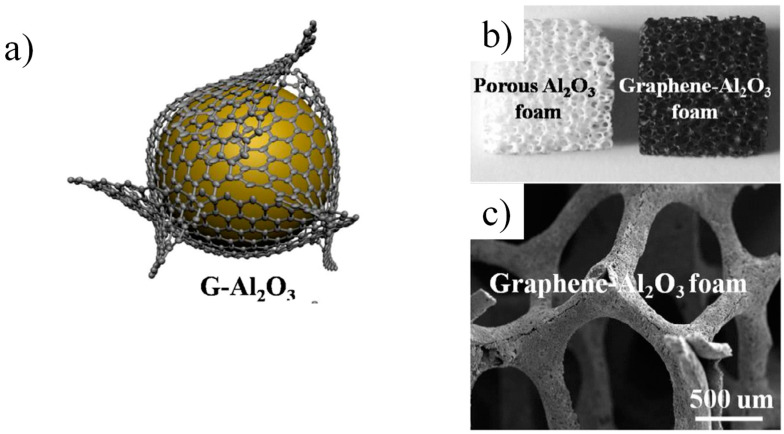
A schematic representation of graphene on Al_2_O_3_ particle (**a**), porous Al_2_O_3_ foam with and without graphene (**b**), and high-resolution SEM image of graphene/Al_2_O_3_ foam (**c**) [140]. Adapted with permission from [140]; published by [Elsevier], (2014).

Extensive research has focused on optimizing the composition, formation, and sintering techniques for graphene-reinforced ceramics, including zirconia (ZrO_2_) [114], alumina (Al_2_O_3_) [115], silicon carbide (SiC) [116], zirconium diboride (ZrB_2_) [117], and boron carbide (B_4_C) [118]. In the case of silicon nitride (Si_3_N_4_), studies have also demonstrated the beneficial toughening effects of graphene [1,107]. These studies, as well as others, highlight that the strengthening and toughening performance of graphene depends on its dispersion, volume fraction, type of ceramic matrix, and processing parameters of the composite [119,120]. Figure 6 represents a typical example of using graphene with alumina.

A variety of applications benefit from the enhanced mechanical and functional properties provided by the addition of graphene [121]. Applications of ceramic/graphene composites are diverse and include energy production and storage, sensors, tissue engineering, electromagnetic interference shielding, thermal management, protection against wear and corrosion, and catalysis [121]. Table 4 lists some of the ceramic/graphene applications.

**Table 4:** Summary of selected ceramics reinforced with graphene, including some examples of their applications in different fields.

Applications	Type of Ceramics and References
Energy production and storage	Li-ion battery cathodes: vanadium pentoxide (V_2_O_5_) [122], cobalt oxide (Co_3_O_4_) [123] Anodes: void-containing Al_2_O_3_/coated porous Si [124] Si_3_N_4_-coated Si core [125],
Piezoelectric energy harvesting	Lead zirconate titanate (PZT) [126], Barium titanate [127]
Sensors	Tin oxide (SnO_2_) [128,129] γ-alumina [130] Zin oxide (ZnO) [131,132]
Electromagnetic interference shielding	Boron carbide (B_4_C) [133] Magnetic iron oxide (Fe_3_O_4_) [134] Nickel cobalt sulfide (NiCoS) [135]
Catalytic applications	Titanium dioxide (TiO_2_) [136] Bismuth vanadate–silicon dioxide (BiVO_4_/SiO_2_) [137] Iron oxide/Nickle oxide (Fe_3_O_4_/NiO) [138]
Heat sinks and thermal energy storage	Alumina (Al_2_O_3_) [139,140] Dual silicon oxycarbide (SiOC) [141,142]

### 4.3. Using Carbon Nanotubes to Increase the Properties of Ceramics

**Figure 7:**
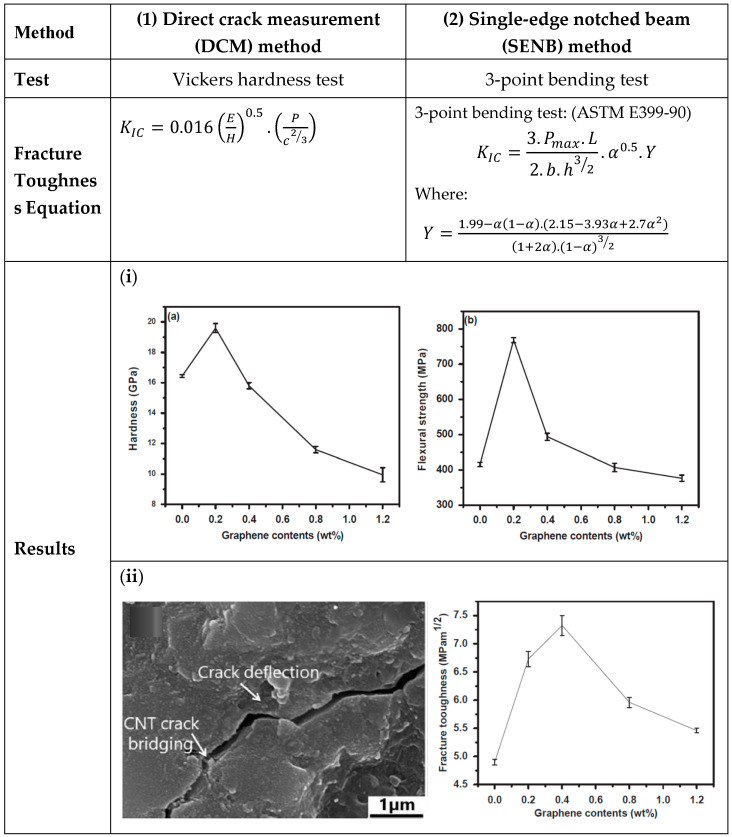
A schematic diagram of the fracture toughness calculation methods. In method one, fracture toughness is calculated by the equation proposed by Anstis et al. [151,152], while equations in method two are based on ASTM E399 [153]. The inset graphs (**i**,**ii**) obtained by Shah et al. [99] represent the results of hardness and flexural strength ((**a**,**b**) in graph (**i**)) and calculated fracture toughness and SEM image of alumina ceramic composites with the addition of 1 wt% carbon nanotubes and 0–1.2 wt% graphene (graph (**ii**)). Inset graphs are reproduced with permission from [99] published by [Elsevier], (2021).

Reinforcing ceramic matrices with carbon nanotubes (CNTs) has gained considerable interest due to the significant improvements CNTs can bring to both the mechanical and functional properties of ceramics. The incorporation of CNTs into engineering ceramic matrices improves the toughness of ceramics, addressing their intrinsic brittleness and improving their fracture resistance [143]. CNTs contribute to toughness enhancement through mechanisms such as crack bridging, crack deflection, and pull-out, which help to prevent crack propagation within the ceramic matrix [144]. These mechanisms significantly improve the durability and mechanical performance of composite materials with different ceramic matrices such as Al_2_O_3_ [144], B4C [145], and ZrB_2_–SiC [146,147]. The toughness of ceramic/carbon nanotube composites can be calculated using two different methods: single-edge notched beam (SENB) method and direct crack measurement (DCM) method [144]. A comparison between the two methods is illustrated in the inset table within the schematic diagram in Figure 7. The inset graphs obtained by Shah et al. represent the fracture toughness of alumina ceramic composites with the addition of 1 wt% carbon nanotubes and 0–1.2 wt% graphene. The results showed that the fracture toughness increased by 49.5% with 1 wt% carbon nanotubes and 0.4 wt% graphene [99].

Beyond mechanical properties, CNTs also improve the functional properties of ceramics, particularly their electrical and thermal conductivities [148,149]. The excellent electrical conductivity of CNTs allows for the creation of ceramic composites with enhanced electrical properties, which are useful in various electronic applications [143]. Similarly, the high thermal conductivity of CNTs enables the development of ceramic composites that can efficiently dissipate heat, making them suitable for applications in thermal management [150]. However, achieving a uniform dispersion of CNTs within the ceramic matrix is crucial for maximizing these benefits. Homogeneous distribution ensures that the reinforcing effects of CNTs are evenly applied throughout the material, preventing areas of weakness and enhancing overall performance [150]. Advanced processing techniques, such as spark plasma sintering and hot pressing, have been employed to achieve better dispersion and bonding of CNTs within ceramic matrices [118].

Applications of CNT-reinforced ceramics are diverse, including structural components, electronic devices, thermal management systems, and more. The combination of improved mechanical toughness and enhanced functional properties makes CNT-reinforced ceramics a promising material for high-performance applications [143].

### 4.4. Combination of Graphene and Carbon Nanotubes

**Figure 8:**
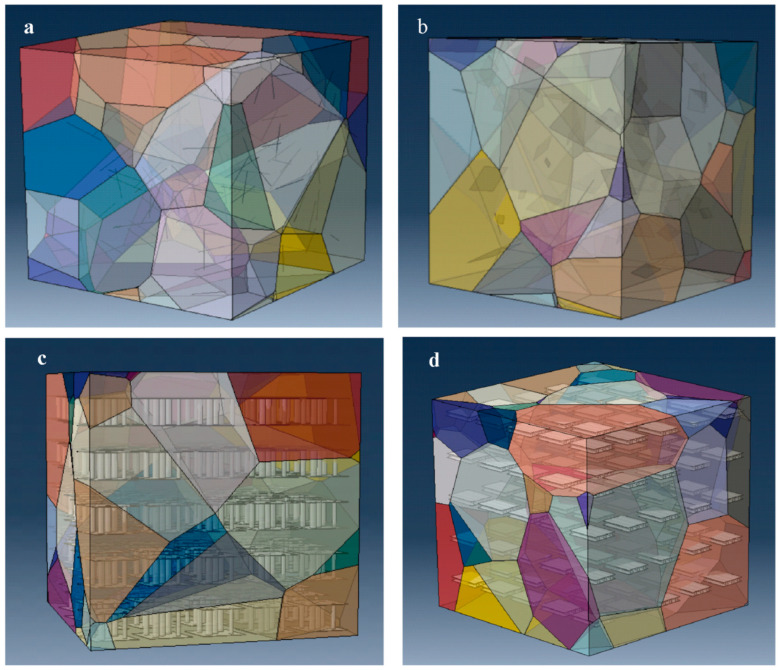
A schematic graphic of random introduction of CNT and graphene into a ceramic matrix. (**a**) Carbon nanotube, (**b**) graphene, and (**c**,**d**) hybrid CNT and graphene [155]. Reproduced with permission from [155]. Published by [Elsevier], (2021).

Combining graphene and carbon nanotubes (CNTs) as reinforcing materials in ceramic matrices has been shown to significantly enhance the properties and performance of ceramics. The synergistic effects of these two materials (mostly in nanosize) enhance both mechanical and functional properties, making them suited to advanced applications in various sectors [154]. Figure 8 displays a schematic diagram of a hybrid ceramic composite consisting of carbon nanotube and graphene and the ceramic matrix represented by a Voronoi element [155].

The combination of these two reinforcing materials, i.e., graphene and CNTs, in ceramic matrices improves fracture toughness through mechanisms such as crack bridging, crack deflection, and necking [99,156,157]. These mechanisms are effective because graphene provides high tensile strength and flexibility, and CNTs contribute to a high aspect ratio and superior mechanical properties [99,158]. This combination influence arises from preventing crack propagation and distributing stress more evenly within the ceramic matrix, thus significantly improving toughness and durability [154,156]. Moreover, the functional properties of ceramics, such as electrical and thermal conductivity, are also enhanced by incorporating both graphene and CNTs. Graphene’s high electrical conductivity and CNTs’ superior thermal conductivity make composites ideal for applications that need efficient heat dissipation and electrical performance [156]. Thus, GNT-reinforced ceramics are ideal for electronic equipment, thermal management systems, and structural parts subjected to high thermal and electrical loads [159].

Various ratios of graphene nanoplatelets to CNTs were used to prepare these composites, providing insights into the optimal composition required for maximum enhancement. For example, it was found that the mechanical properties of Si_3_N_4_ were improved with the addition of up to 5% CNT/graphene content, whereas the ceramic/CNT/graphene composite materials exhibited excellent improvement in electrical and wear properties with a very small quantity (only 1%) of CNT/graphene content [154]. The optimal reinforcement for Al_2_O_3_–TiC composites was obtained at 0.8 wt% MWCNTs and 0.2 wt% GNPs, according to a study performed by Daming Sun et al. [158]. Other properties of ceramics, like the thermal conductivity of monolithic alumina (Al_2_O_3_), were optimized to best values at low CNT and graphene concentrations, which were 0.4 wt% graphene and 1 wt% CNTs [160]. 

Achieving uniform dispersion of graphene and CNTs within the ceramic matrix is crucial for maximizing these benefits [155]. This is due to the fact that increasing the amount of reinforcing materials, i.e., graphene and CNT, leads to agglomeration, which results in the deterioration of the mechanical properties of the composite and limits their usability [155,158]. However, the dispersion of graphene is easier than that of CNT due to the fiber morphology of CNT and the van der Waals interactions between them, which result in nonuniform distribution [99,158]. Furthermore, advanced processing techniques like HP and SPS are employed to ensure homogeneous distribution and strong interfacial bonding. These methods not only enhance the mechanical properties but also preserve the inherent advantages of the nanomaterials [154,158].

In summary, the combined use of graphene and CNTs in ceramic composites results in materials with superior mechanical strength, toughness, and functional properties, paving the way for their application in a variety of high-performance fields. However, achieving uniform dispersion and strong interfacial bonding remains a key challenge that must be addressed through innovative processing techniques and careful material design.

## 5. Challenges of Using Carbon Nanotubes in Ceramics:

(i) Homogeneous Dispersion

**Figure 9:**
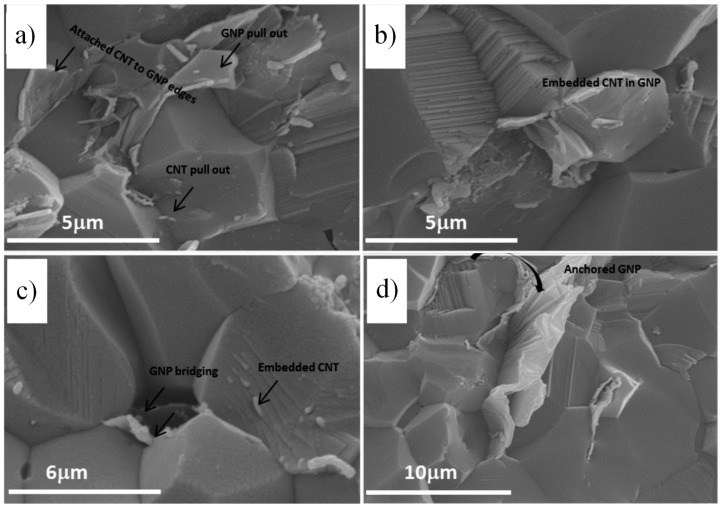
SEM images of the fractured surfaces of hybrid CNT/graphene alumina nanocomposite showing the graphene and CNT pull-out mechanism (**a**), CNTs embedded in graphene flake surfaces (**b**), CNTs and graphene bridging phenomena (**c**), and large graphene rolled along the alumina grain (**d**) [161]. Adapted with permission from [161] published by [Elsevier], (2015).

Hybrid ceramic nanocomposites represent an advanced class of materials that incorporate two or more different nanoscale reinforcements, mainly CNTs and graphene, to improve the mechanical and functional properties of ceramics such as fracture toughness, thermal stability, and electrical conductivity [89,140]. The inclusion of CNTs helps mitigate the brittleness of ceramics by providing mechanisms such as crack bridging, pull-out, and crack deflection, which prevent crack propagation and improve toughness [160]. SEM images in Figure 9 display the dispersion of the hybrid ceramic composite of CNTs, graphene, and alumina in which the pull-out and bridging mechanisms were observed [161].

However, to create ceramic/CNT composites with improved properties, it is crucial to achieve proper interfacial bonding between the ceramic matrix and CNTs and ensure uniform dispersion of CNTs along the grain boundaries. Silvestre et al. [162] reviewed the enhancement of mechanical properties in ceramic composites, emphasizing the role of homogeneous dispersion of CNTs within the ceramic matrix. This is because CNTs often agglomerate due to their high aspect ratio, resulting in stress concentration and subsequent reduction in the overall properties of the composites. This agglomeration is the most significant challenge when preparing CNT–ceramic matrix composites (CMCs). When CNTs bundle or agglomerate, they cause non-uniform distribution within the composites, leading to degradation of fracture toughness, particularly noticeable during indentation analysis. Large amounts of CNT bundles also cause porosity, reducing the contribution of CNTs to the reinforcement mechanism. Uniform dispersion using surfactants and sonication ensures the reinforcing effects of CNTs are optimized, leading to enhanced mechanical strength and toughness [163]. However, surfactantless CNTs were also successfully prepared and homogeneously incorporated within α-alumina ceramics, providing promising reinforcing efficiency [164]. Rubel et al. [165] reviewed the challenges associated with CNT agglomeration in reinforced composites. They noted that achieving a homogeneous distribution of CNTs is essential for optimizing the mechanical properties of CMNCs. Advanced processing techniques, such as sol-gel, hydrothermal/solvothermal, and colloidal processing, are critical for achieving this dispersion [165,166]. Additionally, the in situ growth of CNTs by chemical vapor deposition (CVD) can achieve uniform CNT distribution by growing CNTs at the designed growth sites (metal catalysts) within the ceramic precursor [167].

(ii) Suitable Interfacial Adhesion between CNTs and Ceramics

**Figure 10:**
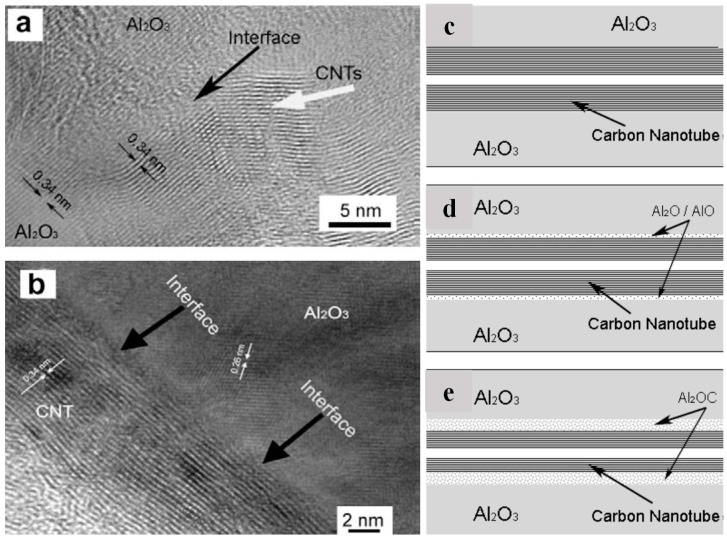
CNT/ceramic interfaces are observed in high-resolution TEM images (**a**,**b**) and illustrated by a schematic model (**c**–**e**) [150]. Adapted with permission from [150]; published by [Elsevier], (2010).

The functional properties of ceramic composites, including their mechanical and thermal characteristics, are highly dependent on the interfacial adhesion between carbon nanotubes (CNTs) and the ceramic matrix. This is because graphene is easier to disperse in ceramic matrices than CNTs [155]; thus, strong interfacial adhesion ensures efficient load transfer from the ceramic matrix to the CNTs, enhancing the composite’s overall strength and toughness. For instance, when the CNT–ceramic interaction is weak, strong phonon scattering occurs, resulting in high thermal resistance, which diminishes the composite’s thermal conductivity [143]. Moreover, weak interfacial adhesion between CNTs and the ceramic matrix can exacerbate crack propagation. This weak bonding fails to effectively utilize toughening mechanisms such as crack bridging, where the CNTs bridge the crack surfaces and impede crack growth, thus improving toughness [113]. Additionally, poor interfacial adhesion undermines the pull-out mechanism, where CNTs are pulled out of the matrix during fracture, dissipating energy and enhancing toughness [156]. Evidence of CNTs sticking to alumina at the interface is shown in the high-resolution TEM images in Figure 10a,b and illustrated in the schematic model in Figure 10c–e [150]. The unknown interface layer shown in Figure 10a,b is suggested to be primarily composed of Al_2_OC after the reaction during the high-temperature and high-pressure sintering process, as illustrated in Figure 10c–e [150].

To overcome these challenges, surface functionalization of CNTs can be employed to enhance interfacial bonding. Techniques such as sol-gel processing and chemical vapor deposition (CVD) can modify the surface of CNTs, increasing their compatibility with the ceramic matrix and improving the overall composite properties [166]. Properly engineered interfacial adhesion not only enhances mechanical properties like fracture toughness but also improves thermal conductivity, making CNT-reinforced ceramic composites suitable for high-performance applications.

(iii) Thermal Degradation of Carbon Nanotubes in Ceramics

**Figure 11:**
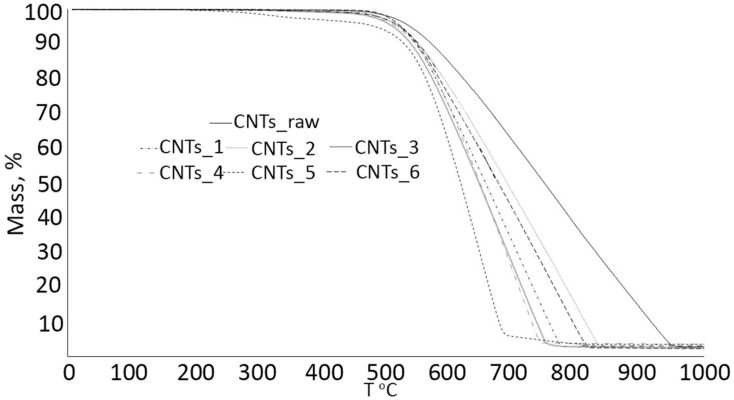
TGA curves of CNTs after SPS processing [169]. Reproduced with permission from [169]; published by [Elsevier], (2020).

Thermal degradation is considered a significant concern in the fabrication of CNT-reinforced ceramic composites. Previous studies have demonstrated that at high pressures, elevated sintering temperatures, and prolonged sintering durations, the structural integrity of CNTs can be influenced [168]. It was found that spark plasma sintering (SPS) at high temperatures can induce phase transformations in CNTs, resulting in a reduction in their mechanical and thermal behaviors [169]. Additionally, the synthesis of ceramic–CNT nanocomposites at high temperatures leads to degradation of CNTs, affecting the overall performance of ceramic composites [170]. To eliminate these effects, optimizing sintering parameters such as temperature, pressure, and duration is essential. Thus, this approach helps preserve the structural integrity of CNTs, ensuring that the ceramic composite material retains its improved mechanical and thermal properties. Thermal degradation of CNTs in ceramic composites by SPS as can be observed by SEM images and Raman spectra [168]. According to a study conducted by F. Inam et al., the SEM images showed that the multi-walled carbon nanotubes in boron carbide composite were damaged and became fibrous, while the Raman spectra revealed that there was severe structural degradation [168]. The decomposition stages of the CNTs under different SPS conditions can be explained by the thermal gravimetric analysis (TGA) test, as shown in Figure 11, based on a study conducted by E. Suslova et al. [169].

## 6. Conclusions

This comprehensive review highlights that engineering ceramics and their composites play a crucial role in modern industrial applications in various areas, thanks to their remarkable properties like high wear and corrosion resistance, low friction coefficient, and structural robustness. By focusing on key materials such as silicon carbide, alumina, zirconia, silicon nitride, and aluminum nitride, the article underscores their versatile applications across industries such as automotive, aerospace, medical, and tribological sectors.

Furthermore, the incorporation of advanced materials like graphene and carbon nanotubes is shown to significantly enhance the prevailing properties of engineering ceramics, including mechanical, thermal, and electrical properties. Hence, graphene and CNT-based ceramic composites are ideal for demanding applications in various high-performance industries.

Below are routes for improving the key properties of engineering ceramics by the inclusion of nano or micro-sized second-phase materials:

(1) The inclusion of carbon nanotubes (CNTs) as a reinforcing phase has been shown to enhance both the toughness and functional properties, such as electrical and thermal conductivity, of ceramic matrices.
(2) Graphene nanoplatelets are another effective reinforcing material. They improve the fracture toughness of ceramics through mechanisms like crack deflection, bridging, and pull-out, leading to increased mechanical performance.
(3) Hybrid composites, combining different reinforcing materials such as CNTs and graphene, can further enhance the properties of ceramics, providing a balance of strength, toughness, and other functional attributes.

Additionally, various preparation methods, including ball milling and spark plasma sintering, are critically analyzed to offer insights into optimizing ceramic properties for specific applications. This comprehensive review not only consolidates current knowledge but also paves the way for future research and development aimed at harnessing the full potential of engineering ceramics in cutting-edge technological advancements.

## Figures and Tables

**Table 1:** Typical properties of high-density α-alumina with 99.99 wt% content [48].

Property	Alumina (Content: 99.99 wt%)
Density, g/cm^3^	3.97–3.99
Melting point, °C	2054
Ultimate strength, (flexure (σf)) MPa	282
Compression, MPa	2550–3100
Modulus of elasticity, GPa	366–410
Crack resistance (K_1c_), MPa·m^0.5^	2.8–4.5
Hardness: Vickers (HV), GPa	19.3
Thermal conductivity at room temperature, W/(m·K)	38.9
Thermal expansion coefficient (10^−6^/K) at 200–1200 °C	6.5–8.9
Specific volumetric electrical resistivity (ρ), Ohm·m	2 × 10^14^
